# Determination of Aluminium and Physicochemical Parameters in the Palm Oil Estates Water Supply at Johor, Malaysia

**DOI:** 10.1155/2010/615176

**Published:** 2011-03-07

**Authors:** M. R. Siti Farizwana, S. Mazrura, A. Zurahanim Fasha, G. Ahmad Rohi

**Affiliations:** ^1^Environmental Health Programme, Faculty of Allied Health Sciences, The National University of Malaysia, 50300 Kuala Lumpur, Malaysia; ^2^Health Risk Assessment Unit, Environmental Health Research Centre, Institute for Medical Research, 50588 Kuala Lumpur, Malaysia; ^3^Department of Biomedical Sciences, Faculty of Allied Health Sciences, The National University of Malaysia, 50300 Kuala Lumpur, Malaysia

## Abstract

The study was to determine the concentration of aluminium (Al) and study the physicochemical parameters (pH, total dissolved solids (TDS), turbidity, and residual chlorine) in drinking water supply in selected palm oil estates in Kota Tinggi, Johor. Water samples were collected from the estates with the private and the public water supplies. The sampling points were at the water source (S), the treatment plant outlet (TPO), and at the nearest houses (H1) and the furthest houses (H2) from the TPO. All estates with private water supply failed to meet the NSDWQ for Al with mean concentration of 0.99 ± 1.52 mg/L. However, Al concentrations in all public water supply estates were well within the limit except for one estate. The pH for all samples complied with the NSDWQ except from the private estates for the drinking water supply with an acidic pH (5.50 ± 0.90). The private water supply showed violated turbidity value in the drinking water samples (14.2 ± 24.1 NTU). Insufficient amount of chlorination was observed in the private water supply estates (0.09 ± 0.30 mg/L). Private water supplies with inefficient water treatment served unsatisfactory drinking water quality to the community which may lead to major health problems.

## 1. Introduction

Safe drinking water is an essential need for human well-being, health, development, and necessity, and therefore, it is internationally recognized as fundamental human rights [1]. 

In Malaysia, drinking water is managed on a state-by-state basis to cater different demand in supply either by public or private water supply. According to Engineering Services Division (ESD) of the Ministry of Health (MOH) [2] public water supply is systematically treated supplied by the government or legislative authorities. Meanwhile, private water supply is defined as water supply which is not provided by the legislative authority and the maintenance relies on the owner of the area; typically in rural areas. The high installation cost of water pipes resorted the owners to supply the population with the nearest water sources [3, 4]. The provision of water by nongovernment authorities often raise water quality issues as the private owners always fail to meet the drinking water quality standard hence initiated outbreaks and health risks as studied by Melian et al. in Republic of Moldova [5], Reid et al. in Scotland [6], and Said et al. in England and Wales [7].

This is due to the water source itself that contributed contaminants where water treatment system done by the above mentioned party was insufficient and unsatisfactory. Contaminated water sources had caused by surrounding activities such as industrial metal mining, according to Huang et al. in his study in Tibet [8]. In another study in Tibetan Plateau, Huang et al. found out that the four major Asian rivers recorded the presence of Mg, Al, Fe and Pb contributed by the mining and agricultural activities, domestic sewage, traffic density [9] as well as the climate change factor [10]. 

Inappropriate treated water may expose the vulnerable water to contaminants such as microbes [11], heavy metals [12], pesticides [13] and pathogens which could lead to water-borne disease outbreaks [14] and ill-health.

The water quality monitoring done by Ministry of Health Malaysia in agricultural areas in Peninsular Malaysia showed violation of water quality standard for several physical and chemical parameters such as pH, turbidity, residual chlorine, aluminum, arsenic and lead [4]. 

In Kota Tinggi, Johor, palm oil estates constitute majority of the district land in agriculture. A total of 28 out of 42 estates in Kota Tinggi still depend on water supply treated and provided by the private estates management [15].

One of the heavy metal contaminants of great concern is aluminium (Al). Al is the most abundant element and occurs naturally by several mobility factors in the environment as silicates, oxides and hydroxides. Excessive addition of Al salts as coagulants in water treatment process might produce elevated concentrations of aluminum in finished water. Al salts are used to reduce organic matter, colour, turbidity and microorganism levels [16].

Human exposure to high Al levels by oral exposure affects a large number of health components as reported by a number of studies in various countries. Those exposures originated from water in drinks and food preparation, cooking utensils [17] as well as antacid preparation [18]. These studies had hypothesized that Al is the risk factor of Alzheimer's disease (AD) [19, 20], dementia, osteomalacia (OM) [21], encephalopathy [22] and total parenteral nutrition (TPN) [23].

Exposure to Al can be determined in human by biological samples such as blood, urine, hair, brain and bone according to the interest of the study. Both urinary and plasma aluminum levels reflect body burden and current exposure but urinary concentration is more responsive according to Nieboer et al. [17] which this finding is in agreement with WHO [18, 24] which reported that urine is the most important route of aluminum excretion.

Acknowledging the limited knowledge of Al exposure among consumers of private water supply and realizing the fact that excessive Al content is detrimental to health, it is the interest of this study to assess the Al concentration in drinking water consumed by this community. Hence, the data obtained from the study can serve as evidence to the estates management specifically to improve the quality of water supply provided and generally to the policy makers in policy development for 100 percent safe water supply in rural agricultural areas.

This paper describes the quality of water supply in selected palm oil estates in Kota Tinggi in terms of heavy metal levels and physicochemical characteristics during one year study period. 

## 2. Materials and Methods

### 2.1. Sampling Site

The study was conducted in selected palm oil estates in Kota Tinggi, Johor. Kota Tinggi which is located to the southeast of Peninsular Malaysia, about 350 km from the capital of Malaysia, Kuala Lumpur. Kota Tinggi is the largest district in Johor with a total of 3500 km² land area [25] populated by 192,336 people [26]. In Kota Tinggi, palm oil plantation is the major activity besides other agricultural and fishery activities.

**Figure 1:**
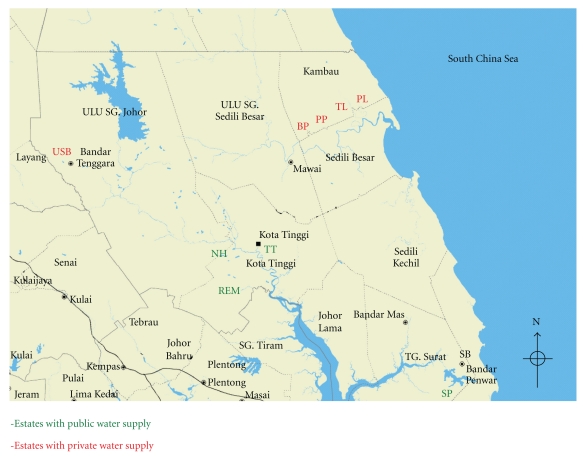
Map of Kota Tinggi, Johor, Malaysia. *Source: e-Map Johor Darul Takzim, First Edition, Department of Survey and Mapping Malaysia (2009) *[22].

For this study, the selected estates consisted of six estates with private water supply and four estates with public water supply. The criteria for selecting those estates considered the type of water supply used in particular estate. Only resided estates with either type of water supply were chosen (private or public water supply only). A few estates with mixed water supply (both type of supplies) were excluded from this study.  Figure 1 illustrated the sampling sites for this study. 

### 2.2. Sample Collection

The water sampling was conducted for one year period with the bimonthly frequency of sampling, from January 2009 to January 2010.

The sampling points for private water supply were at the source (S), treatment plant outlet (TPO), the nearest house from the TPO (H1) and the furthest house from the TPO (H2). While for the public water supply, the same sampling method was used with the exclusion of source (S).

Water from the tap at the TPO, H1 and H2 were let to flush for five minutes to clear off any precipitation. The collection of water samples was carried out according to protocols and instructions to avoid contamination during sampling, storage and transportation.

For the determination of Al levels, water samples were collected in 1 litre polypropylene Nalgene bottles thoroughly cleaned by soaking in 5% nitric acid for 48 hours and rinsed repeatedly with ultra pure water prior to sampling. Upon collection, 2 ml of 69–70% nitric acid was added to the water samples for preservation. Then, the samples were filtered and kept at 4°C until further examination where all samples were thawed prior to the laboratory analysis.

As for the *in situ* measurements, water samples were collected in a clean beaker and 10 ml glass vials were rinsed with the water sample itself before each measurement for every sampling point.

### 2.3. Methods and Analysis

Analysis of Al in water samples was performed by Perkin Elmer ELAN 9000 Inductively Coupled Plasma-Mass Spectrometry (ICP-MS) according to Mahar et al. [27] with modifications. Instrument operational conditions were adjusted to yield optimal determinations as shown in Table 1.

The measurements for pH, TDS, turbidity and residual chlorine were done *in situ* using HACH CO150 pH Meter, HACH Sension5, HACH 2100P Turbidimeter and HACH Spectrophotometer DR 2010, respectively, according to the HACH instruments manual. All the instruments were calibrated accordingly before each fieldwork.

#### 2.3.1. Water Samples Preparation for Al Analysis

Water samples with visible suspended solids or noticeable turbidity were filtered through 0.45 *μ*m membrane filter prior to analysis. All water samples were allowed to reach room temperature before being analysed. Water samples were directly injected into the ICP-MS through the sample tube for measurements, without any dilution.

#### 2.3.2. Water Samples Preparation for Physicochemical Analysis

Water samples for pH, TDS and turbidity measurements were directly measured with the instruments until the values appeared on the reading screen without adding any reagent into the water samples. Meanwhile, for residual chlorine determination, a specific reagent was added into every water sample in a glass vial and shaken for approximately 20 seconds before placing in the spectrophotometer for measurement.

#### 2.3.3. Calibration Solution

The ICP-MS calibration for Al analysis in water samples was using the 10 mg/l multi element standard 3 stock, prepared to 1.0, 5.0, 10, 25, 50, 100, 200, and 500 *μ*g/l concentration of calibration solution.

#### 2.3.4. Quality Control

For the purpose of quality control of the ICP-MS performance while analyzing water samples, the quality control check was conducted for every six samples with 25 ppb calibration solution.

The accuracy of the measurements of Al in water was assured by standard reference material 1643e (Trace Elements in Water).  Table 2 showed the certified values and achieved values for the quality control of the instrument.

#### 2.3.5. Statistical Analysis

All statistical tests were estimated at 95% level of confidence. All data were analyzed with SPSS 16.0 using parametric and nonparametric test to test for differences between variables and correlation analysis to explore the relationship between variables.

## 3. Results

The Malaysian National Standard for Drinking Water Quality (NSDWQ) by the MOH (2004) [28] was used as comparison to interpret the results for Al concentrations in water samples. The maximum acceptable value for Al in water is 0.2 mg/L.

The pH acceptable value ranged from 5.5 to 9.0 for raw water and 6.5 to 9.0 for drinking water. The TDS acceptable value in the standard is 1500 mg/L for raw water and 1000 mg/L for drinking water. The highest acceptable turbidity value for raw water is 1000 NTU while for drinking water is 5 NTU. Meanwhile, the residual chlorine in water sample must be not less than 0.2 mg/L according to the NSDWQ. 

### 3.1. Aluminum in Water

A total of 207 water samples were analyzed for Al concentration.  Table 3 showed the mean and standard deviation of Al concentrations from each estate for both private and public water supplies. According to the table, all estates with private water supply violated the NSDWQ acceptable value. The highest Al concentration was found in estate SB with a mean of 1.59 ± 1.87 mg/L. The overall mean concentration of Al in estates with private water supply was 0.99 ± 1.52 mg/L.

On the contrary, Al concentrations in all estates with public water supply were well within the NSDWQ limit except for estate SP which slightly violated the standard with the mean concentration of 0.41 ± 0.22 mg/L. A lower mean concentration of Al was found for the entire estates with public water supply which was 0.12 ± 0.20 mg/L. A significant difference between the Al concentration in private and public water supply was obtained from the statistical analysis (*P* < .05). 

### 3.2. Physicochemical Quality of the Water Samples

A total of 214 samples were analysed for pH measurements with two categories of water samples. The 36 samples served as raw water while the remaining 178 samples as drinking water. For raw water samples, only estate PP/BK and estate TL complied with the pH limit of the NSDWQ with mean of 5.94 ± 0.51 and 5.65 ± 0.41, respectively. As for drinking water in private water supply, only estate PL complied with the NSDWQ for pH with mean pH of 6.85 ± 0.31. Unlike private water supply, all public water supply estates were having mean pH values within the standard. The statistic analysis showed a significant difference in mean pH between the estates (*P* < .05). A significant weak negative correlation was found between Al and pH levels (*r* = −0.3, *P* < .05).

TDS measurements were done using 36 raw water samples and 170 drinking water samples with a total of 206 water samples. Observed, none of the estates violated the NSDWQ for TDS.

A sum of 213 samples were analysed for turbidity. A number of 36 and 177 water samples served as raw and drinking water, respectively. It was found that only drinking water samples from the private water supply failed to meet the standard. Out of six private water supply estates, only estate PL was having a good mean turbidity value of 1.32 ± 1.02 NTU. A statistically significant difference in turbidity was found between the estates.

A total of 163 drinking water samples were collected for residual chlorine analysis. None of the estates in private water supply were having adequate residual chlorine in their water supply. Similarly, estates with public water supply were having low mean residual chlorine concentration except for estate SP (0.6 ± 0.21 mg/L). The residual chlorine levels for both estates showed a significant difference (*P* < .05) with higher mean levels in PUB.

Table 4 summarized the mean values and standard deviation for each physicochemical parameter according to the estates and water sample categories.

### 3.3. The Noncompliances

**Figure 2:**
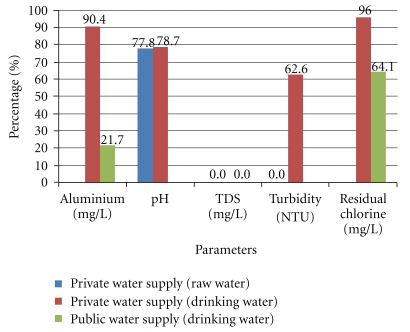
The noncompliance percentages of the water samples for Al and physicochemical parameters according to the estates.

Figure 2 illustrated the noncompliance percentages of the water samples to NDWQS for Al and physicochemical parameters according to the estates. Private water supply was having high percentage of noncompliances for Al (90.4%), pH (77.8% for raw water; 78.7% for drinking water), turbidity (62.6%) and residual chlorine (96.0%), while public water supply violated the Al and residual chlorine standard with noncompliance percentages of 21.7% and 64.1%, respectively.

## 4. Discussion

Aluminium is widely used in raw water treatment by adding the aluminium sulphate (alum) in coagulation and flocculation processes to ensure safe drinking water for human consumption by minimizing the levels of organic matter, microorganism, colour and turbidity [29, 30].

In this study, Al concentration in treated water (TPO, H1 and H2) was higher than the Al concentration at water source (S). This is due to the excessive amount of alum added during the water treatment process. The addition of alum was done only by observing the water turbidity, not according to the correct calculated required amount of alum. As surveyed, the estates management did not hire a certified water treatment operator but basic-trained personnel to handle the water treatment system.

Moreover, the concentration of Al in water is determined by pH level where by high acidity results in corrosion of piping structure and may cause partial solubilization of the element in the water distribution system [31].

According to the results obtained in this study, the turbidity between raw and drinking water samples in private water supply showed slight reduction but still violated the standard. Throughout this trend, it is acknowledged that the water treatment process is not efficient to supply clean water for the estate residents. 

The study has provided insights into the quality of drinking water in terms of aluminium concentrations for private and public water supplies in selected palm oil estates in Kota Tinggi, Johor. From this study, it was observed that private water supply exhibited a lower quality of drinking water which is unsatisfactory compared to the public water supply for the heavy metal quality as well as the physicochemical quality. 

## 5. Conclusion

The inefficiency of the water treatment system may pose a health risk to the community. It is recommended that the estate management to take appropriate actions to improve the water treatment and regular monitoring to be carried out. The health status of the estates residents need to be monitored and more frequent drinking water quality surveillance should be performed by the health authorities.

## Figures and Tables

**Table 1:** Optimization file data of ICP-MS.

Description	Value
Nebulizer gas flow	0.63 L/min
Lens voltage	6.00 Volts
ICP RF power	1000.00 Watts
Analog stage voltage	−2350.00 Volts
Pulse stage voltage	1350.00 Volts

**Table 2:** Certified values and achieved values for SRM 1643e.

Elements	Units	Certified	Achieved	Percent of recovery (%)
Aluminium	*μ*g/L	141.80 ± 8.60	147.59 ± 44.85	104.08
Arsenic	*μ*g/L	60.45 ± 0.72	58.14 ± 6.68	96.2
Cadmium	*μ*g/L	6.57 ± 0.07	6.28 ± 1.50	95.6
Chromium	*μ*g/L	20.40 ± 0.24	23.2 ± 3.92	113.7
Lead	*μ*g/L	19.63 ± 0.21	19.57 ± 3.78	99.7

**Table 3:** The mean and standard deviation concentration (mg/l ± standard deviation) for aluminium (Al) in estates with private and public water supply.

Estates with private water supply						Estates with public water supply			
PP/BP	PL	BK	TL	SB	USB	TT	NH	REM	SP
0.81 ± 1.20	0.52 ± 0.91	1.03 ± 1.39	0.98 ± 1.38	1.59 ± 1.87	1.04 ± 2.08	0.02 ± 0.01	0.03 ± 0.02	0.02 ± 0.01	0.41 ± 0.22
0.99 ± 1.52						0.12 ± 0.20			

**Table:** (a) Mean ± standard deviation for the raw water samples in private water supply

Physicochemical parameters (NSDWQ acceptable value)	Estates with private water supply					
	Raw water					
	PP/BP	PL	BK	TL	SB	USB
pH (5.5–9.0)	5.94 ± 0.51	3.62 ± 0.19	4.90 ± 0.40	5.65 ± 0.41	4.77 ± 0.13	4.12 ± 0.47
TDS (1500 mg/L)	19.30 ± 4.47	42.60 ± 24.72	20.40 ± 4.28	26.00 ± 10.51	25.4 ± 3.48	27.30 ± 12.59
Turbidity (1000 NTU)	22.57 ± 13.54	2.62 ± 1.40	28.30 ± 28.26	13.83 ± 6.70	3.81 ± 4.00	10.03 ± 9.34
Residual chlorine (NA)	No residual chlorine analysis was done for raw water samples					

**Table:** (b) Mean ± standard deviation for the drinking water samples in private water supply

Physicochemical parameters (NSDWQ acceptable value)	Estates with private water supply					
	Drinking water					
	PP/BP	PL	BK	TL	SB	USB

pH (6.5–9.0)	5.53 ± 0.31	6.85 ± 0.31	5.48 ± 0.85	5.60 ± 0.71	5.37 ± 0.69	5.47 ± 0.32
TDS (1000 mg/L)	21.00 ± 2.85	89.20 ± 24.15	36.40 ± 15.94	37.5 ± 16.85	37.50 ± 6.40	46.50 ± 12.14
Turbidity (5 NTU)	19.05 ± 17.57	1.32 ± 1.02	18.65 ± 17.57	10.70 ± 6.84	8.09 ± 5.31	5.48 ± 5.30
Residual chlorine (not less than 0.2 mg/L)	0.07 ± 0.06	0.04 ± 0.03	0.04 ± 0.03	0.03 ± 0.02	0.04 ± 0.08	0.02 ± 0.04

**Table:** (c) Mean ± standard deviation for the drinking water samples in public water supply

Physicochemical parameters (NSDWQ acceptable value)	Estates with public water supply			
	Drinking water			
	TT	NH	REM	SP

pH (6.5–9.0)	8.29 ± 0.34	8.65 ± 0.42	7.84 ± 0.45	7.06 ± 0.18
TDS (1000 mg/L)	54.20 ± 5.05	54.25 ± 4.48	50.91 ± 2.45	27.2 ± 2.54
Turbidity (5 NTU)	0.49 ± 0.24	0.70 ± 0.32	0.55 ± 0.39	1.00 ± 0.21
Residual chlorine (not less than 0.2 mg/L)	0.13 ± 0.05	0.16 ± 0.07	0.14 ± 0.05	0.60 ± 0.21
